# A statistician’s perspective on digital epidemiology

**DOI:** 10.1186/s40504-017-0063-9

**Published:** 2017-11-24

**Authors:** Michael Höhle

**Affiliations:** Department of Mathematics, University of Stockholm, Stockholm, Sweden

**Keywords:** Infectious diseases, Big data, Bias, Interdisciplinarity, Digitalization

## Abstract

We address the question “does digital epidemiology represent an epistemic shift in infectious disease epidemiology” from a statistician’s viewpoint. Our main argument is that infectious disease epidemiology has not changed fundamentally as it always has been data-driven. However, as the data aspect has become more prominent, we discuss the statistical toolbox of the modern epidemiologist and argue that problem solving in the digital age, more than ever requires an interdisciplinary quantitative approach.

## Introduction

The discipline of infectious disease epidemiology is on the move. While globalization has enabled the spreading of infectious diseases at unseen geographic and temporal scales, the digital revolution has generated novel opportunities for the collection and analysis of data. This has lead Salathé et al. (2012) to introduce the term *digital epidemiology*.^1^ The symposium on *Digital Epidemiology and its ethical, legal and social implications* (DELSI) invited participants to answer the question, if “the use of emerging technologies and digital tools, especially *Big Data*, present an epistemic shift in epidemiology” (Eckmanns 2015). In short, the statistician’s answer to this question is: No.

## It’s the data, stupid

Yes, digitalization has brought a range of exciting opportunities, but at the core the discipline of infectious disease epidemiology has not changed: It is about responding to emerging infectious disease threats and controlling endemic diseases in populations by supporting assumptions and actions by *data*. The fact that infectious disease epidemiology is about data has not changed much since Daniel Bernoulli’s smallpox analyses in the 1760’s (Dietz and Heesterbeek 2002).

The variety, volume and velocity of the data may have changed, thus making the discipline more data driven than previously, but the fundamental statistical issues about representativeness and uncertainty in trade-off with practicability, ethics and privacy remain the same. Much of the new data are often collected for other purposes than to be used in epidemiology – Harford (2014) calls this data the *digital exhaust* of our lives. This also means that the much praised big data sources are of poor quality in relation to the epidemiological problem in need of a solution. For this reason, the collection of routine surveillance data by public health institutions targeted at specific problems and question remains crucial. Before tapping new digital data sources, it appears prudent to improve on the shortcomings of the traditional sources by simple – not always digital– means, for example by improving infrastructure and processes to reduce reporting delays, to quantify under-reporting and by supplementing clinical information of cases with adequate microbiologic information, e.g. molecular typing. Another attractive digital option is the fusion of traditional sources (surveillance databases, sentinel networks, questionnaires, food tracing) with novel data sources and forms of communication, e.g., by supplementing a general practioners sentinel surveillance system with a web-based syndromic monitoring system (Bayer et al. 2014).

## Data driven epidemiology

Conducting high quality epidemiology is difficult, if the epidemiologist is not prepared for the discipline being more *data driven*. In what follows we therefore discuss the importance of interdisciplinary cooperation and of having an appropriate toolbox to deal with data.

### Filling the toolbox

Terms like “machine learning”, “predictive modelling” and “big data” sound auspicious – in particular if they are accompanied by a promise to solve highly complex epidemiological problems. For a statistician, however, the terms are marketing jargon referring to statistical methods for high-dimensional data analysis covered in any modern text book, e.g., Efron and Hastie (2016). In other words, the bread and butter logistic regression model remains an indispensable machine learning tool even for big data analyses. Statistical extensions of logistic regression modelling are, e.g., concerned with the ability to flexibly handle continuous covariates, investigate more covariates than available data points (aka. *p*≫*n* inference) and the calibration of model parameters with the objective of *predicting* the outcome variable - opposed to *explaining* the outcome variable. As a modern epidemiologist it is worthwhile to know these extensions and the situations where they are helpful, e.g., when many food items are analytically investigated by case-control studies during foodborne outbreaks.

A further important aspect in the transition of epidemiology is that programming and data handling skills have become more essential, in particular when one needs to deviate from standard analysis. This includes the need to reshape large amounts of data, organize data beyond the flat table format, perform natural language processing on unstructed text or create visualizations of multidimensional data as a means of communicating insights. All these tasks go beyond what the average epidemiologist can achieve by manual steps with familiar spreadsheet software. Concurrently, the academic community has started to support its methodological developments by directly providing collaborative open-source software tools implementing the methods, e.g., the modelling and visualization of incidence time series, outbreak detection as well as the estimation of the basic reproduction number during outbreaks (Jombart and FitzJohn 2016; Salmon et al. 2016; Obadia et al. 2016). Even methods for the synthesis of epidemiologic and genetic data are available (Jombart et al. 2014). Methods which, at least from a statistician’s viewpoint, belong to the toolbox of every modern infectious disease epidemiologist. Thanks to the open-source approach these methods are now available with a minimal programming effort.

At present, this digital transition of epidemiology is often not mirrored by a shift of content in education programmes. As an example, the European Programme for Intervention Epidemiology Training (EPIET), educating the new generation of infectious disease epidemiologists in Europe, contains only a small section on data analysis and statistical methods. Visualization, data management and programming skills are essentially left to learning-by-doing. Other aspects, such as software enhanced collaborative analyses (e.g. by revision control), quality assurance as well as reproducibility of analyses remains unknown territory.

### The importance of interdisciplinary cooperation

The need of epidemiologists to simultaneously possess medical and microbiologic insights as well as analytic skills, knowledge on policy development, communication and cultural competency, financial planning and management skills (Birkhead and Koo 2006) underlines that an interdisciplinary approach towards modern epidemiology is needed. This is not new, but in light of the increased demand for analytical skills, even the best training and experiences in data analysis can only get the epidemiologist to a certain point. Beyond this point cooperation with data and data modelling experts is needed. Statisticians are such experts, but they can be frustrating to communicate with: They insistently keep pointing out potential biases of your data and chant that (digital) garbage in results in (digital) garbage out. Furthermore, they demand an investigation of uncertainty related to every derived insight – this includes the aspects of model specification, model fitting, model validation as well as data fusion (Chatfield 1995). Such comprehensive investigations are often in conflict with clear, simple and consistent messages for a paper or press release. Nevertheless, such investigations are necessary, because they provide scientific justification of model based approaches – approaches which are inherent when answering epidemiological questions by data. In summary, the modern epidemiologist has a lot to gain by getting a statistician on board of data driven projects early on.

## Discussion

The above represented a statistician’s perspective on infectious disease epidemiology in a digital age. Issues about representativeness and uncertainty remain crucial especially in the world of big data. All of this is not new, but statisticians do have useful answers to these problems – there is no need to reinvent statistics. The traditional shoe-leather epidemiology remains fundamental, but it is only efficient in a digital world, if supported by a strong analytic back-office.

## Endnote

^1^ In what follows we interpret–given the context of the DELSI symposium–digital epidemiology exclusively as digital *infectious disease* epidemiology.
